# Analysing Intercellular Communication in Astrocytic Networks Using “Astral”

**DOI:** 10.3389/fncel.2021.689268

**Published:** 2021-06-15

**Authors:** Egor Dzyubenko, Wojciech Prazuch, Matthias Pillath-Eilers, Joanna Polanska, Dirk M. Hermann

**Affiliations:** ^1^Department of Neurology and Center for Translational Neuro- and Behavioral Sciences (C-TNBS), University Hospital Essen, Essen, Germany; ^2^Department of Data Science and Engineering, Silesian University of Technology Faculty of Automatic Control, Electronics and Computer Science, Gliwice, Poland

**Keywords:** data analysis, calcium imaging, glia, network coupling, astrocyte-neuron interactions

## Abstract

Astrocytic networks are critically involved in regulating the activity of neuronal networks. However, a comprehensive and ready-to-use data analysis tool for investigating functional interactions between the astrocytes is missing. We developed the novel software package named “Astral” to analyse intercellular communication in astrocytic networks based on live-cell calcium imaging. Our method for analysing calcium imaging data does not require the assignment of regions of interest. The package contains two applications: the core processing pipeline for detecting and quantifying Ca^++^ events, and the auxiliary visualization tool for controlling data quality. Our method allows for the network-wide quantification of Ca^++^ events and the analysis of their intercellular propagation. In a set of proof-of-concept experiments, we examined Ca^++^ events in flat monolayers of primary astrocytes and confirmed that inter-astrocytic interactions depend on the permeability of gap junctions and connexin hemichannels. The Astral tool is particularly useful for studying astrocyte-neuronal interactions on the network level. We demonstrate that compared with purely astrocytic cultures, spontaneous generation of Ca^++^ events in astrocytes that were co-cultivated with neurons was significantly increased. Interestingly, the increased astrocytic Ca^++^ activity after long-term co-cultivation with neurons was driven by the enhanced formation of gap junctions and connexin hemichannels but was not affected by silencing neuronal activity. Our data indicate the necessity for systematic investigation of astrocyte-neuronal interactions at the network level. For this purpose, the Astral software offers a powerful tool for processing and quantifying calcium imaging data.

## Introduction

Investigating intercellular communication in astrocytic networks is essential for understanding how neuronal activity is regulated in health and disease. Although astrocytes do not exhibit neuron-like action potentials, they respond to neuronal stimulation by transiently increasing intracellular Ca^++^ concentration (Schipke and Kettenmann, 2004). Astrocytic Ca^++^ events can be triggered by a variety of stimuli, including local release of neurotransmitters (Haydon, 2001) and paracrine signalling mediated by purine secretion (Haas et al., 2006; Hoogland et al., 2009). Some of the evoked Ca^++^ events remain localised to small astrocytic compartments, such as perisynaptic endfeet, while others spread over the entire cell like waves. The large wave-like Ca^++^ events can further propagate to neighbouring cells through intercellular gap junctions involving the classical IP_3_/Ca^++^ pathway (Sakuragi et al., 2017) or via the release of gliotransmitters like glutamate or ATP through the hemichannels opening to the extracellular space (Orellana and Stehberg, 2014). In astrocytes, both gap junctions and hemichannels are formed mainly by connexin 43 and play a major role in brain physiology (De Bock et al., 2014). In perisynaptic astrocytic processes, Ca^++^ increases are triggered by glutamate release from excitatory synapses via metabotropic glutamate receptor 5 (mGluR5) (Sun et al., 2013; Tang et al., 2015) and by GABA release from inhibitory synapses via GABA_B_ receptor (Kang et al., 1998; Mariotti et al., 2016). In turn, the increase of astrocytic Ca^++^ induces the release of gliotransmitters by glial cells, such as glutamate, D-serine, GABA, and others, which facilitate synaptic transmission (for review see Araque et al., 2014). At the network level, astrocytic Ca^++^ events and gliotransmitter release synchronise neuronal activity (Fellin et al., 2004; Sardinha et al., 2017), thereby contributing to network functioning.

State-of-the-art knowledge about physiological neuron-astrocyte interactions was generated by correlating Ca^++^ dynamics in single astrocytes to neuronal activity. In the meantime, inter-astrocytic communication was recognised to be essential for controlling the activity of neuronal networks on a larger scale, since the disrupted coupling in astrocytic networks leads to epilepsy-like conditions (Bedner et al., 2015; Deshpande et al., 2017). Astrocytic coupling is commonly analysed by dye distribution assays. In this method, biocytin or fluorescent dyes that are injected into a single cell, distributes over the astrocytic network via gap junctions formed by connexin channels (Khan et al., 2016). Although this approach provides valuable information about the permeability of gap junctions and the anatomy of astrocytic networks, it does not offer the possibility to investigate functional interactions between the astrocytes mediated by Ca^++^ signalling.

While the analysis of Ca^++^ events propagation in single astrocytes was significantly improved recently (Wang et al., 2019), a method for analysing intercellular communication in astrocytic networks is still missing. In this work, we introduce the “Astral” software, which allows investigating astrocyte-astrocyte interactions at a population level.

## Methods

### Legal Issues and Animal Housing

Experiments were performed with local government approval (Landesamt für Natur, Umwelt und Verbraucherschutz, Recklinghausen) in accordance to EU directive 2010/63/EU for the care and use of laboratory animals and local institutional guidelines. C57BL/6j (Envigo, Indianapolis, IN, United States) and Rosa-CAG-LSL-GCaMP6f (Jackson Laboratory, Bar Harbor, ME, United States) mice were kept in groups of 5 animals/cage in a regular inverse 12 h light-dark cycle and access to food and water *ad libitum*. All efforts were made to reduce the number of animals in the experiments.

### Cell Cultures

**FIGURE 1:**
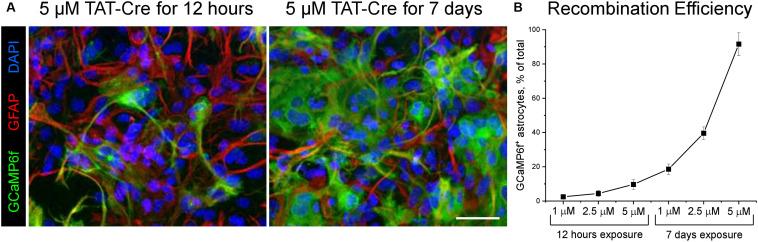
TAT-Cre recombinase enzyme induces robust GCaMP6f expression in astrocytic cultures prepared from the cortices of Rosa-CAG-LSL-GCaMP6f mice. **(A)** Immunocytochemical labelling shows GCaMP6f (green) and GFAP (red) expression in astrocytes treated with 5 μM TAT-Cre for 12 h or for 7 days. Nuclei were counterstained with DAPI. Scale bar, 50 μm. **(B)** Efficiency of genetic recombination induced by 12 h or 7 days of exposure to 1, 2.5, and 5 μM TAT-Cre was quantified as percentage of astrocytes expressing GCaMP6f. Connected squares show means ± SD, *n* = 3.

Primary neuronal and astrocytic cultures were prepared as described previously (Gottschling et al., 2016; Dzyubenko et al., 2017) with minor modifications. Mixed glia cultures were obtained from the cortices of new-born (postnatal day 0–1) Rosa-CAG-LSL-GCaMP6f mice (C57BL/6j background) by gentle dissociation followed by 7 days of cultivation in glia-selective medium [Dulbecco Modified Eagle’s Medium (DMEM) containing 4.5 g/l glucose and 10% v/v fetal bovine serum] at 37°C and 5% CO_2_. After astrocytic monolayers reached confluence, oligodendrocyte precursors and microglia cells were eliminated by rotary shaking (250 rpm) in DMEM medium containing 20 μM cytosine-1-β-D-arabinofuranoside (AraC). The obtained pure astrocytic cultures were further incubated for 7 days in serum-free medium. To induce robust and long-lasting expression of GCaMP6f calcium indicator in the cultivated astrocytes, 5 μM of cell-permeant nuclei-targeted TAT-Cre recombinase enzyme (#SCR508 MilliporeSigma, Burlington, United States) was added to the cultivation medium. The astrocytes were detached with 0.05% trypsin in 0.53 mM EDTA and sub-cultivated (50,000 cells/cm^2^) on poly-D-lysin coated 35 mm dishes (μ-Dish #81156, Ibidi, Gräfelfing, Germany). Before calcium imaging, astrocytes were cultivated for 21 days in Neurobasal medium containing 2% B27 supplement, 100 U/ml penicillin and 0.1 mg/ml streptomycin, allowing for the maturation of astrocytic networks. The mature cultures exhibited 92 ± 6.7% recombination efficiency, as indicated by GCaMP6f immunolabelling (Figure 1), which enabled the analysis of astrocytic Ca^++^ events on the population level.

For evaluating the impact of astrocyte-neuron interactions on Ca^++^ events in astrocytic networks, neuronal cultures were obtained from the hippocampi of C57BL/6j embryos at 15.5 days post conception by gentle dissociation. Neurons (25,000 cells/cm^2^) were plated together with the sub-cultivated astrocytes onto the poly-D-lysin coated 35 mm dishes on the same day. The co-cultures of neurons and astrocytes were maintained for 21 days in fully defined Neurobasal medium containing 2% B27 supplement, 100 U/ml penicillin and 0.1 mg/ml streptomycin, allowing for the maturation of both neuronal and astrocytic networks. In a subset of experiments, neurons (25,000 cells/cm^2^) were co-cultivated with astrocytes on polyethylenimine/polyornithine coated multiple electrode arrays (60MEA200/30iR-Ti, Multi Channel Systems, Reutlingen, Germany) for neuronal network activity evaluation.

### Immunolabelling Procedures

For immunocytochemistry, cell cultures were fixed with 4% w/v paraformaldehyde (PFA) for 15 min at room temperature (RT). Expression of GCaMP6f was verified using rabbit anti-green fluorescent protein antibody (1:300, ab290, Abcam, Cambridge, United Kingdom). Glial fibrillary acidic protein (GFAP) was detected by rat anti-GFAP antibody (1:1,000, 13-0300, ThermoFisher, Waltham, MA, United States). Connexin channels were identified with rabbit anti-connexin 43 antibody (1:500, 3512, Cell Signaling Technologies, Frankfurt, Germany). For fluorescence detection, secondary antibodies conjugated to Alexa or Atto dyes were used. Nuclei were counterlabeled with DAPI (1:1,000, D1306, ThermoFisher).

### Connexin Expression Quantifications

After immunofluorescent labelling, the number and size of connexin 43 (CNX43) clusters was quantified in the single plane 145 × 145 × 5 μm confocal images obtained with the LSM 780 confocal microscope (Carl Zeiss, Jena, Germany) using the 20× Achroplan objective. The images were binarised (automated Otsu threshold selction method) and analysed with the Particle Analyser plugin in ImageJ. The results were expressed as the number of CNX43 clusters per astrocyte (which were detected by the characteristic GFAP expression and elongated nuclei shape) and their size.

### Drugs and Treatments

The role of astrocytic network coupling in Ca^++^ event propagation was evaluated by blocking connexin channels with 20 μM carbenoxolone disodium (CBX, 3096, Tocris, Bristol, United Kingdom). Network-wide calcium elevations were induced by adding 1 μM adenosine to the cultivation medium during calcium imaging. To evaluate the impact of neuronal activity on astrocytic Ca^++^ events, we blocked Na^+^ channels with 1 μM tetrodotoxin (TTX, 1078, Tocris).

### Neuronal Network Activity Recordings

In co-cultures of neurons and astrocytes, neuronal network activity was evaluated by multiple electrode array (MEA) electrophysiology (MEA2100, Multi Channel Systems). Neuronal network activity was recorded for 15 min before and after TTX application. Neuronal activity was analysed and visualised using MC Rack software (Multi Channel Systems). TTX application completely supressed neuronal activity evaluated by MEA.

### Calcium Imaging

Ca^++^ events were observed in GCaMP6f expressing astrocytic networks using a customised AxioPhot (Carl Zeiss) upright epifluorescence microscope (40× W N-Achroplan 40×/0.75 M27 objective) equipped with the Axiocam 702 mono sCMOS camera for live cell imaging and the Colibri 5 light source. In a subset of experiments, wild-type astrocytes were loaded with 2 μM Fluo-3 calcium sensor (Fluo-3 AM, F1241, ThermoFisher) in PBS + 1% Pluronic F-127 for 30 min at 37°C. For each sample, 3 areas (560 × 356 μm) were recorded for 3 min at the rate of 5 frames (960 × 608 pixels, 0.586 μm pixel size) per second using the Zen Blue software (Carl Zeiss).

### Astrocytic Calcium Event Analysis

For the analysis of population level astrocyte-astrocyte interactions, we developed new software called ‘‘Astral.’’ Astral is a tool for analysing and interpreting intercellular communication based on live-cell calcium imaging experiments. As such experiments produce complex data, Astral was created for detection, segmentation, and analysis of calcium oscillations in microscopic time-lapse image stacks. The software is freely available on GitHub^1^. Installation guidelines and user manual are provided in Supplementary Manual.

The Astral application was created with the help of two workflow programming platforms: Airflow^2^ and Streamlit^3^. After installing Astral, the Airflow based processing pipeline will become available under http://localhost:8080/ and the Streamlit based visualization tool will be accessible at http://localhost:8501/ using a web browser. Please note that for using the visualization tools, it is necessary to perform a part of processing pipeline first.

**FIGURE 2:**
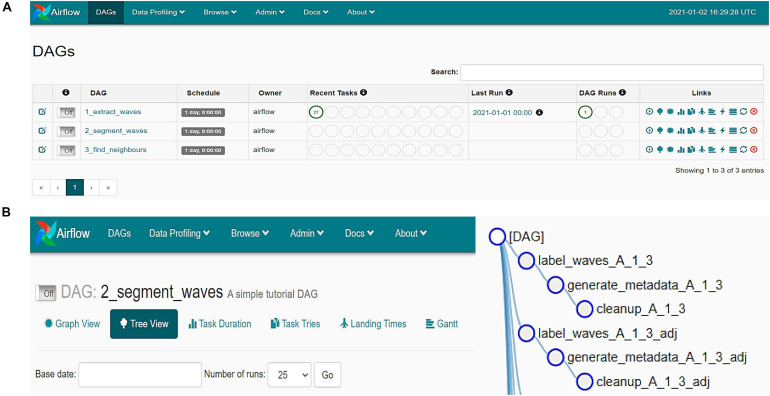
Screen captures demonstrating the Airflow based user interface for the Astral pipeline. **(A)** General overview of the three processing steps. **(B)** Tree-view of the sub-tasks.

The Astral pipeline consists of three processing steps, which are designed as Directed Acyclic Graphs (DAGs). Each of the DAGs comprises a set of tasks, which can be sequentially activated to verify intermediate results and allowing for easier troubleshooting (Figure 2).

The current version of Astral incorporates three groups of tasks:

(1) Extracting calcium events
• *Create timelapse*. This task converts user data into the scientific Numpy array format and saves it for further usage. Currently, only the Tiff format conversion is supported, in which consecutive microscopic images are saved as image series.
• *Extract events*. This step handles the search and extraction of the regions associated with the calcium events. Mean grey value and standard deviation are calculated for a group of pixels at a defined position over time. The number of pixels in a group is defined by the *Grain_Size* variable. Then, based on the standard deviation threshold (*SD_threshold* variable), defined by the user, the pixels are either assigned to a calcium wave, or treated as a background and set to zero. Thereby, a time lapse sequence of binary images is generated is saved as a Numpy array and stored as the waves.npy file.
• *Create masks*. This task groups adjacent non-zero pixels into one single calcium event in 3D space (x, y, and time dimensions). Thereby, each pixel of the image stack is assigned to its calcium event, which has a unique identifier (ID). In this, an optional 3D watershed algorithm can be implemented by setting the *Use_watershed* variable as 1. Please note that the Watershed algorithm requires significant computational resources and can result in the task abortion on weaker computers. The task outputs are labelled arrays, which are saved as “labelled_waves.npy” and “black_and_white.tiff” files.
(2) Segmenting calcium events
• *Label events.* The algorithm gets the coordinates of each pixel of a given calcium event and temporarily saves the “waves_inds.pck” matrix-like object.
• *Generate metadata.* This task creates a tabular data frame object to store a coordinate of each pixel of the given calcium wave together with its intensity. For each calcium wave, the extreme dimensions (maximum xy, maximum t), and Euclidean distances to the centers of mass of all other detected calcium waves are calculated.
• *Cleanup.* The task deletes temporary files and removes the noise associated calcium events which are smaller than *Volume_threshold* voxels.
(3) Finding neighbour calcium waves
• *Find neighbours.* At this step, a boundary box is created around each calcium event. The box dimensions are determined by the user as the *Tolerance_xy* and *Tolerance_t* variables, which define the distance between the box frame and extreme points of the calcium event in the corresponding dimensions. The center of the boundary box is located at the Euclidean center of the calcium event. Any other calcium event located within the box space is assigned as a neighbour. This task produces the “neighbours.csv” file, which lists the IDs of all the neighbours for a given calcium event. This file also includes basic information about the neighbour events (center-to-center distances, extreme xy and t dimensions, etc.) and can be opened in any table editing software (e.g., Excel). The task saves an additional file “neighbour_statistics.csv,” which summarises the average values.
• *Find repeats.* This algorithm extends the previous step: for each group of neighbours, it checks for intersections between them. If the projection of an event ID_n_ onto the xy plane intersects with the projection of its neighbour by more than *Intersection_threshold* value, the neighbour is considered as a repeat of the event ID_n_. As a result, the task assigns a “single” category for single calcium events, and “repeat” category for repeated calcium events. Temporary “repeats.pck” and “singles.pck” files are generated.
• *Generate csv.* This task generates two output csv files describing the properties of repeated and single calcium events (dimensions, inter-repeat time distance etc.)

The full list of user-defined input variables and their default values (optimised for the experiments performed in this study) are provided in Table 1. Please note that for both input variables and the output files, Astral uses pixels and frames as units, which can be recalculated into microns and seconds based on the imaging settings.

**TABLE 1:** Input variables.

**Variable**	**Description**	**Default**
*Filename*	File names of image sequences in the data directory to be analysed (“All:” process the entire batch)	All
Intersection*_threshold*	How much should the calcium events overlap in their z-projection to be treated as repeats	0.8
*Grain_size*	How many adjacent pixels are taken into account for standard deviation calculation (2^n^ format)	1
*SD_threshold*	Standard deviation threshold for detecting calcium events (multiplies of mean value)	5
*Tolerance_t*	A maximum distance in time between subsequent calcium waves considered as neighbours (frames)	5
*Tolerance_xy*	A maximum xy distance between two adjacent calcium waves considered as neighbours (pixels)	50
*Use_watershed*	Shall a watershed algorithm for splitting adjacent calcium events be implemented during mask generation? (0—NO; 1—YES)	0
*Volume_threshold*	Volume threshold in total number of voxels to filter away the noise-associated calcium events. Waves smaller than this value will be excluded	40

It is possible to override the default parameter values. These values are loaded from the variables.json file, located in the parent directory of the application. Modifying the values inside the variables.json file will change the default values of the application from the next startup.

**FIGURE 3:**
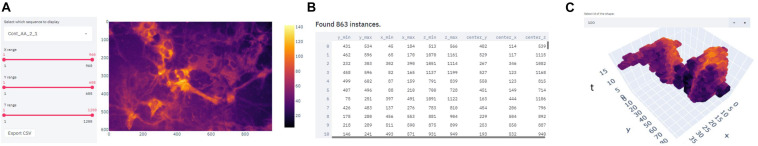
Screen captures demonstrating the Streamlit- based user interface for astrocytic calcium event visualization. **(A)** Selecting the desired position on the time-lapse. **(B)** Tabular overview of the detected waves properties. **(C)** 3D visualization of a chosen event.

To offer a simple but powerful tool for visualising and verifying the quality of processed data, we developed a complementary application using the Streamlit platform. Using the Streamlit-based application, it is possible to navigate through the time-lapse sequence of calcium imaging data (Figure 3A), which simplifies associating a detected Ca^++^ event with its location and morphological properties (Figure 3B). By plotting the 3D shape (x,y,t) of a chosen astrocytic Ca^++^ event, the distribution of intracellular Ca^++^ concentration over time can be examined (Figure 3C), which is particularly useful for studying the dynamics of Ca^++^ events and for customising the user-defined settings.

### Statistics

For non-normally distributed datasets, data were evaluated by Kruskal-Wallis tests followed by pair-wise Mann-Whitney *post hoc* comparisons. For normally distributed datasets, data were evaluated by two-way ANOVA and *post hoc* two-tailed independent Student’s *t*-tests. For multiple comparisons, Bonferroni correction was applied. Statistical analysis was performed using OriginPro2020 software (Origin Lab, Northhampton, MA, United States).

## Results

**FIGURE 4:**
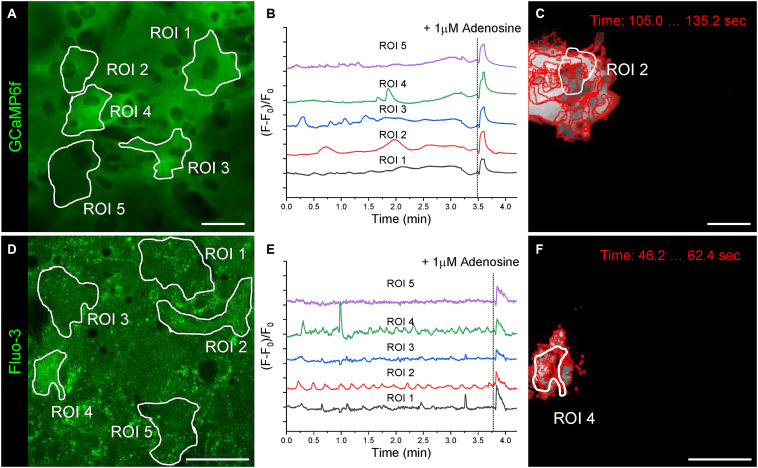
Spontaneous and induced Ca^++^ events in primary astrocytes. **(A)** Astrocytes expressing the genetically encoded calcium sensor GCaMP6f after 21 days of cultivation. White outlines indicate regions of interest (ROIs) that were selected for fluorescence intensity over time analysis based on putative cell borders detected by phase contrast microscopy. **(B)** Fluorescence over time traces (F–F_0_)/F_0_ are shown for the five ROIs selected in **(A)**. **(C)** Ca^++^ event boundaries detected by the Astral software at consecutive time points (grain size 4 pixel, threshold SD*5) is overlaid with the corresponding ROI outline. **(D)** Astrocytes loaded with the calcium sensor Fluo-3 after 21 days of cultivation. ROIs are outlined in white. **(E)** Fluorescence over time traces (F–F_0_)/F_0_ are shown for the five ROIs selected in **(D)**. **(F)** Ca^++^ event boundaries detected by the Astral software at consecutive time points (grain size 4 pixel, threshold SD*2) is overlaid with the corresponding ROI outline. Scale bars, 50 μm.

After 21 days of cultivation in the fully defined serum-free medium, the mature astrocytes spontaneously generated Ca^++^ events and demonstrated transient network-wide Ca^++^ elevations after extracellular stimulation with 1 μM adenosine (Figure 4). The spontaneous Ca^++^ events spread over adjacent cells or localised to a fragment of an astrocyte. In astrocytic networks, the pre-defined regions of interest (ROIs) based on putative cell borders do not reliably represent the key parameters of astrocytic Ca^++^ events. The Astral processing pipeline does not require detection and assignment of cell-specific ROIs, making the analysis of Ca^++^ signalling in astrocytic networks more reliable and unbiased. Instead, Ca^++^ events are detected when fluorescence intensity in a group of pixels (defined by the “grain size”) exceeds the corresponding standard deviation threshold (defined by the “SD threshold”). The adjustable grain size and SD thresholds allow for the detection of Ca^++^ events in both low-noise (astrocytes expressing GCaMP6f, Figures 4A–C) and high-noise (astrocytes loaded with Fluo-3 calcium indicator, Figures 4D–F) conditions.

**FIGURE 5:**
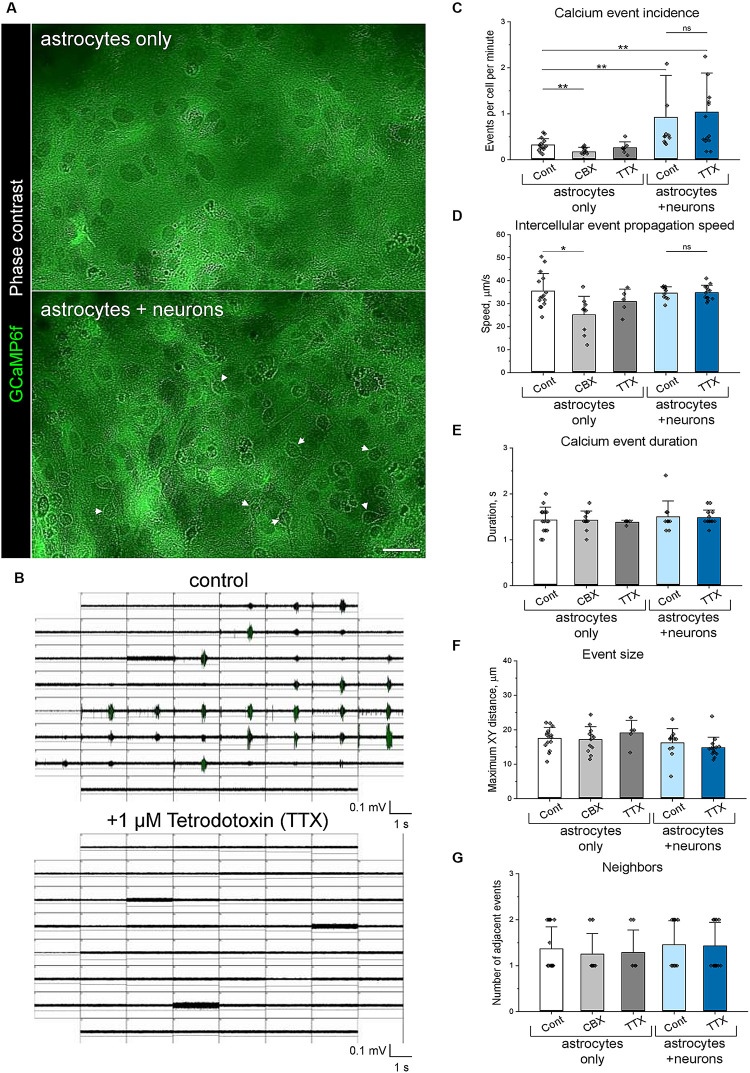
Co-cultivation of primary astrocytes and neurons enhances astrocytic Ca^++^ signalling. **(A)** Live-cell imaging of GCaMP6f expressing astrocytes, which were cultivated with and without neurons. Arrowheads indicate neuronal cell bodies, which can be recognised in phase-contrast images in neuron-astrocyte co-cultures. By preparation, the neurons do not express GCaMP6f. Scale bar, 50 μm. **(B)** Neuronal networks exhibit synchronous activity, indicated by network-wide bursts detected using multiple electrode array (MEA) electrophysiology. Application of 1 μM TTX for 30 min completely silenced neuronal activity. Panels show neuronal activity simultaneously recorded by 59 electrodes (200 μm centre-to-centre distance), each window representing a single electrode. Neuronal activity was measured independently of calcium imaging. **(C–G)** Calcium oscillations in astrocytic networks were analysed using the Astral software, measuring calcium wave incidence **(C)**, median intercellular propagation speed **(D)**, duration **(E)**, distance travelled **(F)** and number of neighbouring waves **(G)**. Bars show mean ± SD, diamonds are data points indicating median values for the recorded area. **p < 0.01, *p < 0.05, based on Mann-Whitney tests. *n* = 5.

Using the Astral software, we quantified astrocytic Ca^++^ events detected during 3-min recording periods in 560 × 356 μm areas (3 per every culture dish) containing 101 ± 40 (mean ± SD) astrocytes. To evaluate intercellular communication, we analysed Ca^++^ event incidence by measuring the number of detected events per cell per minute, intercellular wave Ca^++^ event propagation speed by measuring median time required to travel the centre-to-centre distance between neighbouring events, duration and size of single events, and the number of consequent Ca^++^ events induced in the neighbourhood of a particular event (Figure 5). Of note, measuring intercellular event propagation speed as the time required to travel the centre-to-centre distance between neighbouring events provides a cell-border independent metric for Ca^++^ event propagation analysis.

### Carbenoxolone Impairs Ca^++^ Signalling in Astrocytic Networks

**FIGURE 6:**
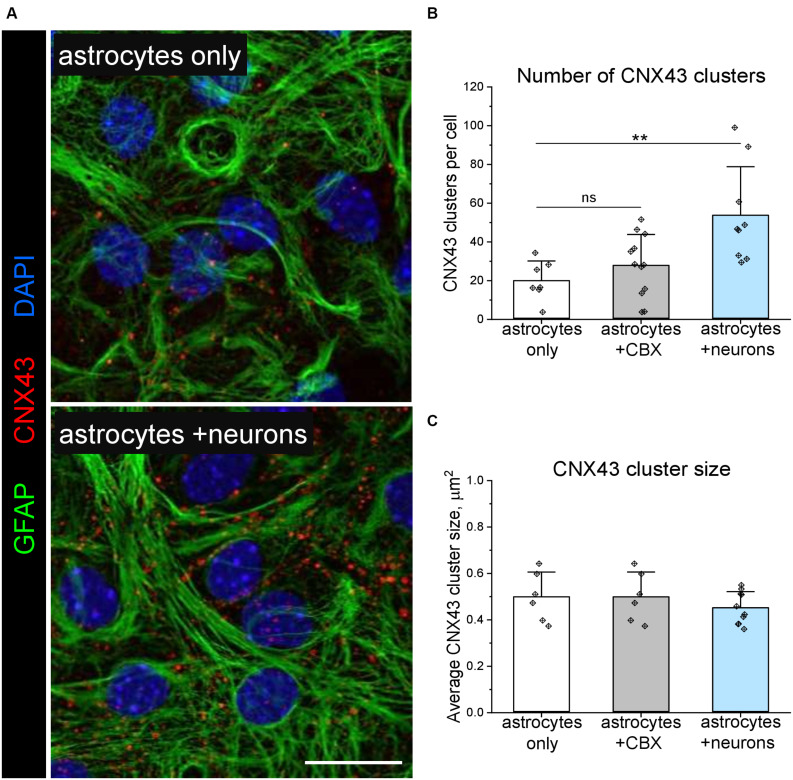
Expression of gap junctions and hemichannels formed by connexin 43 in astrocytic networks cultivated with and without neurons. **(A)** Representative immunolabelling of glial acidic fibrillary protein (GFAP, green) and connexin 43 (CNX43, red) in pure astrocytes, and astrocytes co-cultivated with neurons. Nuclei are labelled with DAPI (blue). Scale bar, 20 μm. Quantification of CNX43 cluster density **(B)** and size **(C)** is shown. Bars show mean ± SD, diamonds are data points. **p < 0.01, based on two-way ANOVA and *post hoc* two-tailed independent Student’s *t*-test. *n* = 5.

In a proof-of-concept study, we used the Astral software to analyse how interastrocytic communication is influenced by the permeability of connexin channels. The application of 20 μM carbenoxolone disodium (CBX) for 30 min, which inhibits both gap junctions (Ma et al., 2018) and connexin hemichannels (Ye et al., 2009), significantly decreased Ca^++^ events incidence (Figure 5C), intercellular propagation speed (Figure 5D), but did not affect the duration (Figure 5E), size (Figure 5F), and the number of neighbouring Ca^++^ events (Figure 5G) in the astrocytic networks. Of note, the observed effects were not associated with a different number of gap junctions/connexin hemichannels per astrocyte, indicated by a similar pattern of connexin 43 (CNX43) expression (Figures 6B,C). Hence, decreasing the permeability of connexin channels with CBX reduced intercellular Ca^++^ event propagation speed and incidence. We hypothesise that the reduction of intercellular Ca^++^ event propagation speed was associated with the inhibition of gap junctions, which could introduce a latency at the border between the cells. The reduced Ca^++^ event incidence is potentially associated with the inhibition of connexin hemichannels.

### Neurons Enhance Ca^++^ Signalling in Astrocytic Networks

To investigate how astrocyte-neuronal interactions influence intercellular communication in astrocytic networks, we compared spontaneous Ca^++^ events in pure astrocytes and in co-cultures of neurons and astrocytes. After 21 days of cultivation, both neurons and astrocytes established mature networks, as indicated by synchronous neuronal activity detected by MEA (Figure 5B) and network-wide astrocytic Ca^++^ events detected by calcium imaging (Supplementary Video 1).

Co-cultivation with neurons (Figure 5A) significantly increased Ca^++^ event incidence (Figure 5C) and in the astrocytic networks but did not affect intercellular propagation speed (Figure 5D), duration (Figure 5E), size (Figure 5F) and the number of neighbouring Ca^++^ events (Figure 5G). The enhanced astrocytic Ca^++^ signalling correlated with the increased number of connexin gap junctions/hemichannels per astrocyte, indicated by a higher density of CNX43 clusters (Figures 6A,B) detected in neuron-astrocyte co-cultures. Interestingly, silencing neuronal activity with 1 μM TTX for 30 min (Figure 5B) did not influence astrocytic Ca^++^ events, and both Ca^++^ event incidence and intercellular propagation speed (Figures 5C,D) remained elevated in comparison with purely astrocytic cultures. In pure astrocytic cultures, TTX application did not affect Ca^++^ signalling (Figures 5C–G).

### Extending Functionality of Astral

Upon necessity, the functionality of Astral software can be further extended to fulfil user-specific needs. For example, fluorescence intensity or motion correction might be desirable for *in vivo* multiphoton calcium imaging experiments. Here, we demonstrate the extendibility of Astral by implementing the previously developed algorithms for correcting the background intensity shifts and motion artifacts (Wong et al., 2005; Marczyk et al., 2014) as an additional pre-processing block. In brief, the mean intensity for each time frame is matched with the mean intensity of the preceding frame. For correcting motion artifacts, the field of view is divided into subregions, and the cluster of subregions with low SD and high mean intensity of the signal is selected. This cluster represents reference regions exhibiting no Ca^++^ oscillations. Next, the entire field of view is shifted by the XY vector calculated from the special shift of the refence. The input and the output of this processing are shown in Supplementary Video 2. For the interested readers, we provide a brief Supplementary Developers Manual.

## Discussion

Growing evidence indicates that astrocytic Ca^++^ signalling is critically involved in neuronal activity regulation (Pirttimaki et al., 2011; Sasaki et al., 2014; Poskanzer and Yuste, 2016; Deemyad et al., 2018). Previously, several methods for analysing local Ca^++^ events in astrocytes were developed, involving compartmentalised Ca^++^ signal quantification (Di Castro et al., 2011; Srinivasan et al., 2015; Agarwal et al., 2017), automated ROI detection (Francis et al., 2012), and volumetric Ca^++^ activity quantification (Bindocci et al., 2017; Savtchouk et al., 2018) in individual astrocytes. Although we know that functional coupling via gap junctions in astrocytic networks allows for the intercellular propagation of Ca^++^ events (Fujii et al., 2017) and is required for preventing overexcitation of neuronal networks (Bedner et al., 2015; Khan et al., 2016), network-wide communication between astrocytes has so far not been systematically studied.

Analysing Ca^++^ events in multiple connected astrocytes offers a compelling possibility of integrative understanding of complex neuron-glia interactions in the brain. Without ready-to-use analytical tools, performing such experiments is difficult. Here, we developed the novel Astral software, which provides a powerful and straightforward pipeline for processing calcium imaging data and for evaluating intercellular communication in astrocytic networks. In comparison to the existing tools offering the analysis of Ca^++^ signalling in single astrocytes (Wang et al., 2019) and neuronal networks (Giovannucci et al., 2019), our approach does not require the assignment of pre-defined ROIs, making network-wide Ca^++^ event detection more flexible and unbiased. To associate Ca events detected with our method with the positions of individual cells, additional cell labelling techniques can be implemented, such as dye loading (Káradóttir and Attwell, 2006) or fluorescent protein expression (Sakaguchi et al., 2018).

The Astral solution contains two applications: the core processing pipeline for detecting and quantifying Ca^++^ events, and the auxiliary visualization tool for controlling data quality. Both utilities were created using a community-driven, open-source Docker virtualization platform application programming interface, which allows for further developing and broadening the applicability of the Astral software. New components of Astral, such as fluorescence intensity and motion correction, can be added without producing conflicts with the earlier ones, making our solution flexible to adjust to a user’s needs.

In a set of proof-of-concept experiments, we applied our tool for analysing Ca^++^ events in flat monolayers of primary astrocytes and confirmed that intercellular interactions in astrocytic networks depend on the permeability of connexin channels. Our method imposes no technical limitations for investigating astrocytic Ca^++^ events *in vivo* by using multiphoton microscopy. Detecting Ca^++^ events in large blocks of intact brain tissue while preserving sub-cellular resolution is challenging because of the restricted field of view and scanning speed of conventional two-photon (2P) microscopes. However, major advances were recently made in the field, significantly improving both scanning speed and size of tissue blocks observable by intravital 2P microscopy (Prevedel et al., 2016). We therefore expect that our analytical tools will be applied in the near future for investigating intercellular communication in large astrocytic networks in living animals using sensory stimulation and local neurotransmitter application approaches.

The unique feature of our approach is quantification of intercellular Ca^++^ signalling. In comparison to other methods (Wang et al., 2019), the Astral software measures not only morphological parameters of single Ca^++^ events such as travelled distance and duration, but also evaluates intercellular propagation of Ca^++^ events between neighbours in astrocytic networks. In this work, we did not implement individual cell labelling to detect the borders between astrocytes, and a part of the events that we defined as neighbours could potentially occur in the same cell. However, this limitation applies only to *in vitro* experimentation in astrocytic monolayers, because in the brain, astrocytes occupy non-overlapping territories (Bushong et al., 2002; Halassa et al., 2007). Therefore, astrocytic Ca^++^ events detected *in vivo* can be reliably attributed to single cells.

Our method is particularly useful for studying astrocyte-neuronal interactions on the network level. In addition to paracrine signalling involving multiple adjacent cells (which we mimicked in this work by adding adenosine extracellularly), astrocytes exhibit more intricate neighbour-to-neighbour communication via localised Ca^++^ events. To investigate the influence of neuronal activity on this type of astrocytic interactions, we co-cultivated primary neurons and astrocytes in fully defined medium for 21 days to allow for the maturation of both cell types. Compared with purely astrocytic cultures, the occurrence of Ca^++^ events in astrocytes that were co-cultivated with neurons was significantly increased. Previous studies demonstrated that neuronal activity induces astrocytic Ca^++^ events within seconds after stimulation (Tang et al., 2015; Mariotti et al., 2016). In this work, we show that silencing neuronal activity by applying TTX for 30 min does not have an immediate effect on the network-wide incidence and propagation of astrocytic Ca^++^ events. After silencing neurons, the occurrence of Ca^++^ events in astrocytic networks remained as high as when neuronal networks were active. We therefore suggest that the increased astrocytic Ca^++^ activity after long-term co-cultivation with neurons can be driven by the enhanced formation of gap junction channels or connexin hemichannels, which mediate network coupling and paracrine signalling, correspondingly. The distinct roles of gap junctions and hemichannels remains to be further elucidated experimentally. Also, we cannot exclude that the effect of neuronal activity silencing unfolds on a different time scale, within a few seconds or, on the contrary, several hours after TTX application.

These data indicate the necessity for systematic investigation of astrocyte-neuronal interactions at the network level, including the analysis of Ca^++^ activity in larger cell populations *in vivo*. The Astral software offers a powerful and expandable tool for studying functional intercellular astrocytic communication by calcium imaging.

## Data Availability Statement

The original contributions presented in the study are included in the article/Supplementary Material, further inquiries can be directed to the corresponding author/s.

## Ethics Statement

The animal study was reviewed and approved by Landesamt für Natur, Umwelt und Verbraucherschutz, Recklinghausen.

## Author Contributions

ED, JP, and DH designed the study. WP developed and optimised new software. ED, MP-E, and WP performed the experiments and analysed the data. ED drafted the manuscript. All authors discussed the results and contributed to the final manuscript.

## Conflict of Interest

The authors declare that the research was conducted in the absence of any commercial or financial relationships that could be construed as a potential conflict of interest.

## Footnotes

1 https://github.com/wprazuch/Astral

2 https://airflow.apache.org/

3 https://streamlit.io/
