# Supplementary material for: Analysing Intercellular Communication in Astrocytic Networks Using “Astral”
Source: Front Cell Neurosci. 2021 Jun 15;15:689268. doi: 10.3389/fncel.2021.689268 (PMC8239356; doi:10.3389/fncel.2021.689268)
Supplement: Supplementary file 1 [file Data_Sheet_1.DOCX]

Astral – User Manual

Contents

[Introduction 2](#_Toc66816434)

[Overview 2](#_Toc66816435)

[Installation and prerequisites 2](#_Toc66816436)

[Building the Astral application 4](#_Toc66816437)

[Running the Astral application 5](#_Toc66816438)

[Astral components and their usage 6](#_Toc66816439)

[Airflow application 6](#_Toc66816440)

[Overview 6](#_Toc66816441)

[Pipeline 7](#_Toc66816442)

[Usage 9](#_Toc66816443)

[Airflow configuration 12](#_Toc66816444)

[Streamlit application 18](#_Toc66816445)

[Overview 18](#_Toc66816446)

[Usage 19](#_Toc66816447)


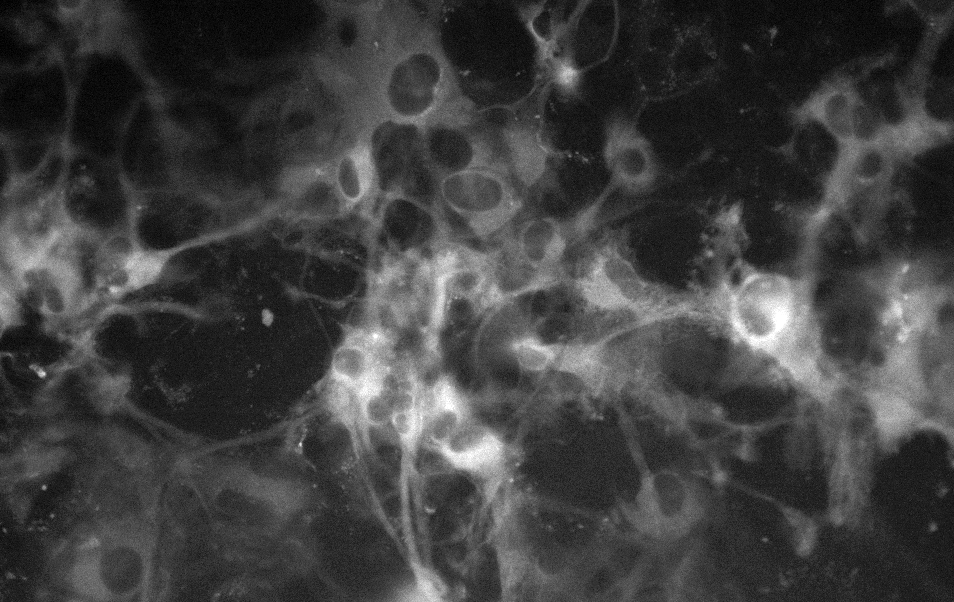


# Introduction

## Overview

Astral is a tool for analyzing and interpreting calcium signalling from a microscopic timelapse imaging. The goal of Astral is to create an easily extensible system for such data. As such experiments produce complex data, Astral was created for detection, segmentation, and analysis of calcium events inside timelapse data. Astral was created in collaboration between the Department of Data Science and Engineering of the Silesian University of Technology in Gliwice and the NeuroScienceLab of the University Hospital Essen.

# Installation and prerequisites

To run Astral, a Docker Desktop application is required. To download Docker Desktop, visit: <https://www.docker.com/products/docker-desktop>

After the installation process, you can launch Docker Desktop, and in the task bar, you should see Docker Desktop’s icon.


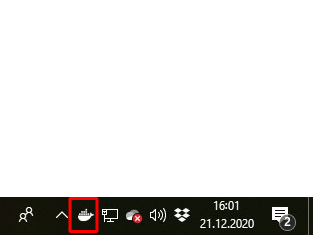


Right-click on it and navigate to **Settings**.


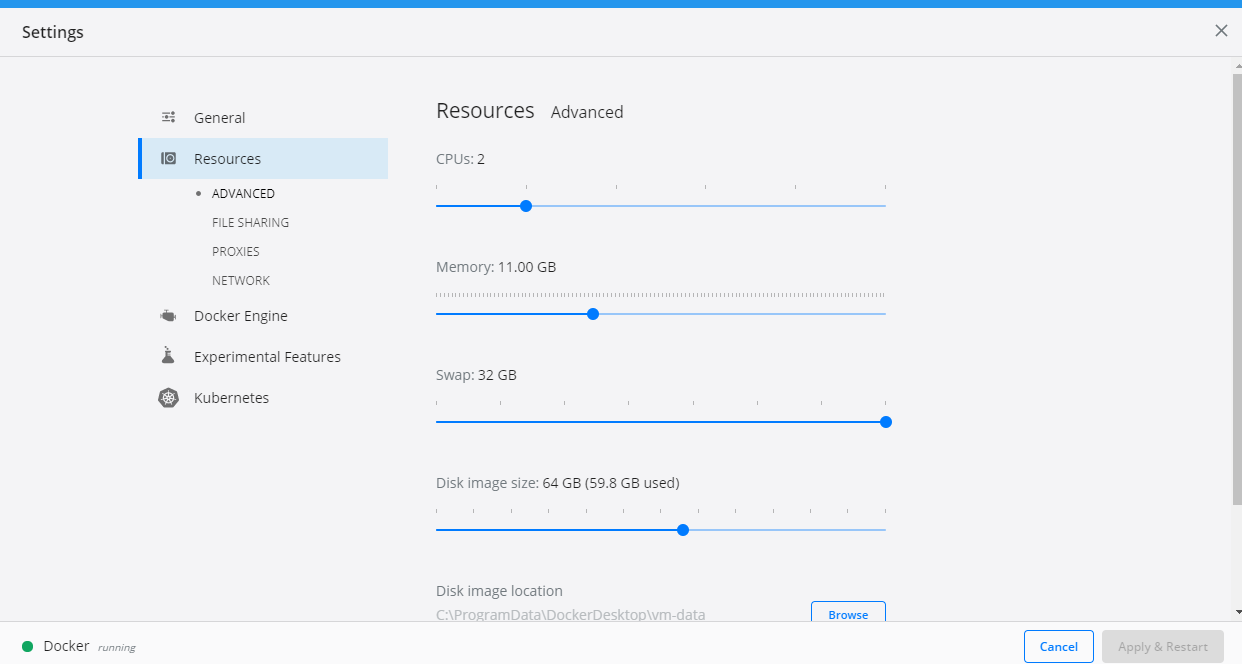


In the **Resources** tab, there are specifications of the computational power that will be dedicated to Docker applications. You may manually customize, how many/much CPUs, Memory, Swap and Disk size you will attribute to Docker during runtime.

For Astral to run properly, a significant amount of Virtual Memory is necessary. If possible, set Memory to at least 10GB. For Swap, set it to 32 GB. Swap is a memory which is used when the application runs out of RAM. Because the timelapse data can be resource-consuming, it is advised to set it as high as possible.

-------------------------------------------------------------------------------------------------------------------------

Hint 1

If the Resources tab is not visible in the Docker panel, it is necessary to install Hyper-V service. The dialog window from the Docker Desktop should appear with the steps to install Hyper-V. In other case, visit:

<https://docs.microsoft.com/en-us/virtualization/hyper-v-on-windows/quick-start/enable-hyper-v>

Hint 2

If you are unable to set Swap to higher values, open up Windows Run window and type:

%appdata%

An explorer will show with an AppData folder. Then, navigate to the following file in the explorer:

AppData\Roaming\Docker\setting.json


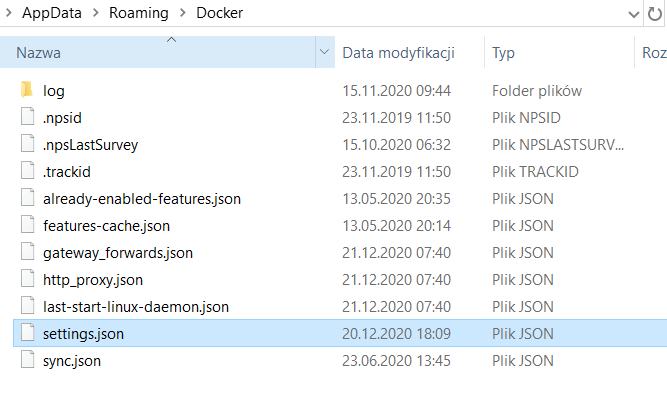


And open it with your favorite editor. Then, to set the swap memory manually, find the swapMiB property:


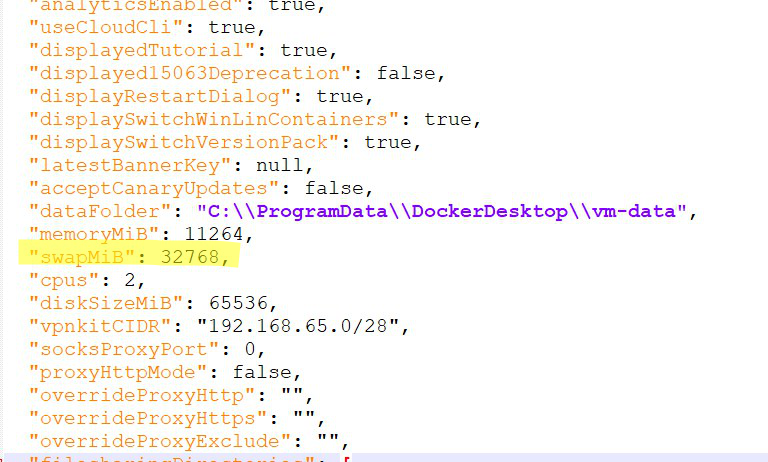


And set it to a desired value.

-------------------------------------------------------------------------------------------------------------------------

Depending on assigned RAM memory amount, swap can be set empirically. For image series of 700 MB size and 8 GB of RAM, a reasonable value for swap is 32 GB, although the required value may be much smaller for some files. The amount of memory depends on the number of events in the image series. This memory is released after application termination, so memory assignment is temporary.

Warning

Remember that swap is used when the program runs out of RAM memory. If the program will need to allocate resources in the swap, but there will not be enough swap memory, the task will crash. In case the step crashes, the first thing to check is to increase swap memory and re-run the task.

## Building the Astral application

To run the Astral application, it is necessary to build it first. **This step is done only at the first start and does not need to be repeated,** unless the software version or data directory for image series is changed. In case the data directory is moved to another location, it is necessary to modify the paths in the docker-compose.yml file.

The first thing to do is to edit the **docker-compose.yml** file, which is located in the parent directory of the application. The file can be opened using any text editor. The file specifies the internal configuration of the application. It consists of many values, which should not be modified, but some values are required to be customized. One of such values is the path to the data directory, in which the image series files will be stored.


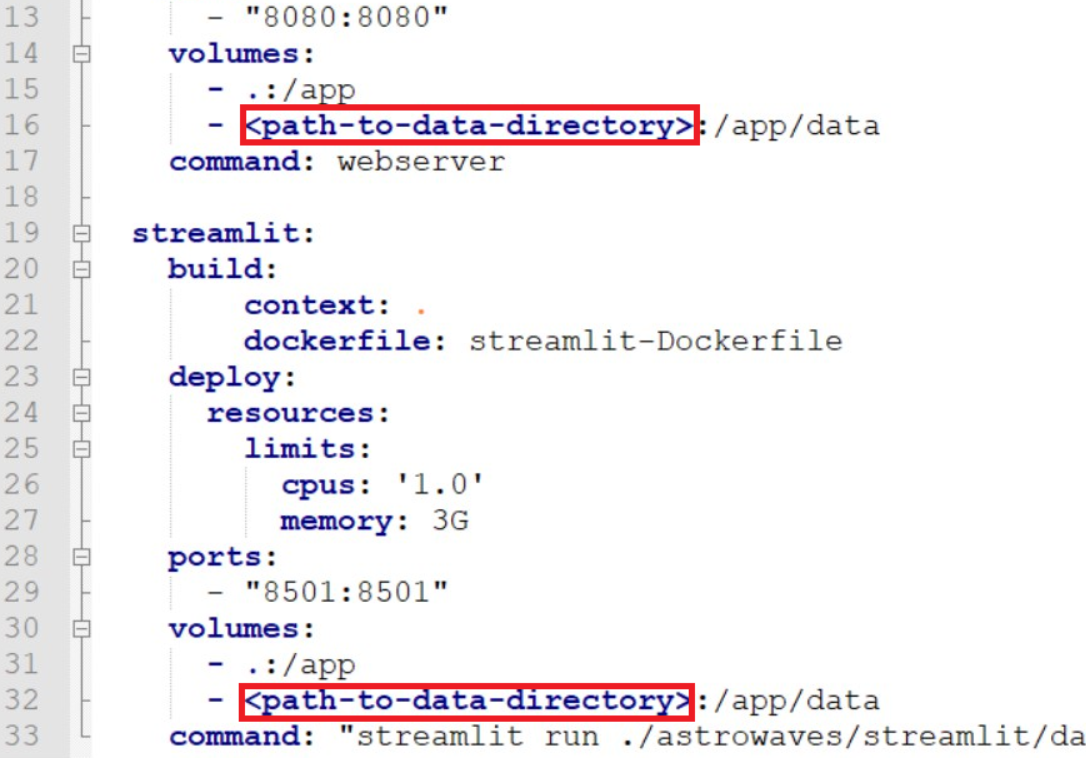


Enter the path to the data directory, which contains the files to be processed by Astral. The path needs to be entered in two places (line 16 and line 32). **Do not remove the *:/app/data part* from the lines.**

To build the Astral application, please follow the steps:

1. Make sure Docker Desktop application is running, and enough RAM and swap memory is assigned;
2. Run the Windows PowerShell terminal;
3. Inside the Powershell command prompt, navigate to the Astral parent directory. To do this, type:

cd “<path-to-Astral-parent-directory”

For example:

cd “C:\Users\user\Documents \Astral”

If the command was executed successfully, a path specified from the command should be shown next to the cursor;

1. Astral application needs to be built – for that to happen, type:

**Docker compose build**

The application building process will start. The process can take up to several minutes, until the command prompt will be available for typing again.

## Running the Astral application

Make sure that the Docker Desktop application is running and launch the Windows PowerShell terminal. Start the application by typing:

**Docker compose up**

Then, in the Powershell window, the application log files will appear. The running application will have a similar output in the window:


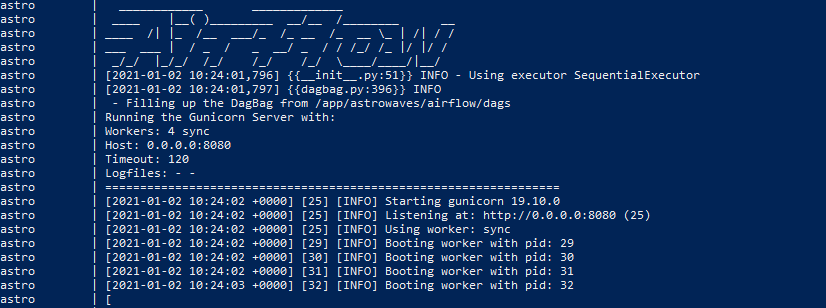


Astral solution consists of two applications, which will become available under two different URLs in the web browser:

- You can access the Airlow application on the URL address: <http://localhost:8080/admin/>
- You can access the Streamlit application on the URL address: <http://localhost:8501/>

Warning

As a prerequisite of using Streamlit application for a given microscopic timelapse image series, it is necessary to perform a part of processing pipeline in the Airflow application

# Astral components and their usage

## Airflow application

### Overview

The Airflow application will be used as a processing pipeline, which performs subsequent steps to generate an analytic output about the data. Some methods present in the Airflow application will be used to generate intermediate data for the next steps as input, whereas other steps will be responsible for end-data generation. Airflow is designed as a framework for data engineering and is a perfect solution for working with scientific data, as requirements and necessities are expected to evolve in the scientific world.

Airflow provides a toolset for defining and creating computer programs which load, handle, transform and generate data, so microscopic timelapse astrocytic events data is a perfect candidate for developing methods of analysis of such data.

Airflow provides a couple of levels of abstraction. For the time being, a notion of Directed Acyclic Graphs (DAGs) and tasks should be introduced. DAG defines a complete set of functionalities which accomplish a specific task. A DAG consists of task – computer programs which are responsible for performing a specific task in the DAG’s workflow. DAG may consist of several tasks, which are connected with each other (mostly sequentially) to generate some final outcome.

Airflow is a perfect option for a community-driven platform – any contributor may create a new functionality and easily incorporate it into Airflow. This may potentially result in a robust, extensive framework, which can be used for many intentions and purposes. Although the initial goal of Astral was to provide a toolset for analyzing astrocytic event data, Airflow makes it possible to develop a toolset for any kind of similar data. This makes Astral a very strong tool in future research in general.


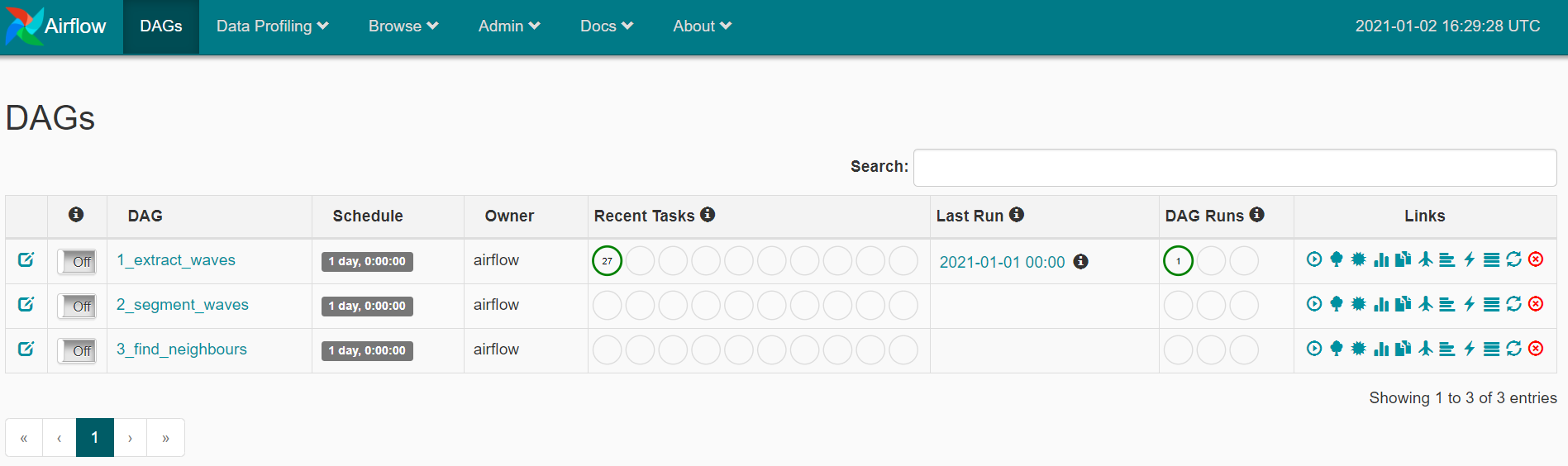


Airflow’s main page displays a list of DAGs available to be executed. By clicking into the DAG name, the website redirects to the DAG’s workflow and execution details:


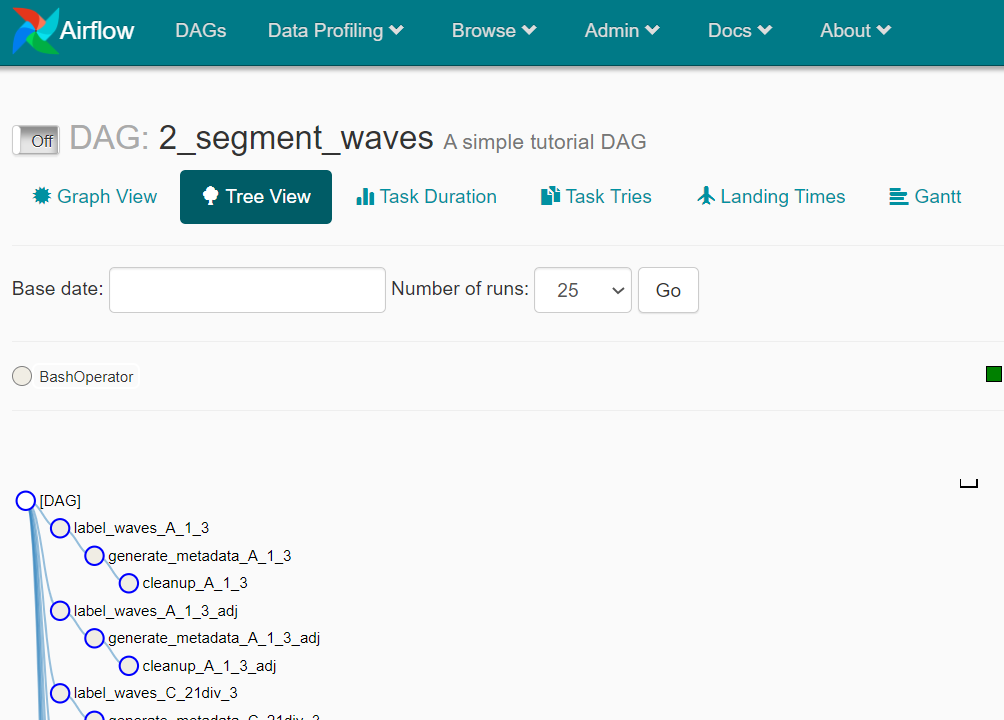


The DAG’s detail page presents a set of tasks, which are needed to be executed for a DAG to finish.

### Pipeline

The processing pipeline is divided into 3 parts: “1_extract_waves”, “2_segment_waves” and “3_find_neighbours”. Each DAG consists of several steps, called tasks, which accomplish a specific operation needed to generate a final output. This architecture is clean and easy to be modified, or extended, so it is easy to extend the functionality of the application.

At this moment, the DAGs are defined by the following tasks:

1. 1_extract_waves:

- Create_timelape – this step deals with converting common data formats into scientific Numpy format, which was created by the creators of numpy, a popular Python scientific package. Currently, only Tiff data format is supported, in which consecutive microscopic images are saved as image series. This step converts such tiff file into a numpy array and saves it for further usage.
- Extract_waves – this step handles search and extraction of the regions associated with the calcium events. A mean and standard deviation for each pixel is calculated in terms of its value across all the image series. Then, based on the standard deviation threshold defined by the user, the pixels are either treated as calcium events or as background.

$T_{ij}=\mu_{ij}+ \alpha\sigma_{ij}$

where i and j denote the positon of the pixel in the image (i represents the x coordinate, whereas j represents y coordinate)

If the pixel has a value greater or equal to the T value, it is considered a calcium wave. In other case, it is considered a background.

All pixels that are considered as background are set to 0. The pixels treated as calcium waves are preserved. The resulting space is saved as Numpy array, as waves.npy file.

- Create_masks – the goal of the task is to group together adjacent calcium wave pixels into one single astrocytic event. In 3D space, using a Volume-like algorithm and 3D connectivity, a single value is assigned to each pixel, which denotes its ID. Additionally, if the use_watershed flag is set, the Watershed algorithm is used in case and two independent calcium waves are connected together. The algorithm’s goal is to detect such cases, and separate the waves.

The task outputs a labelled space, saves as “labelled_waves.npy” file, and “black_and_white.tiff” file, which depicts calcium waves in white, and background in black.

1. 2_segment_waves:

- Label_waves – the goal of the algorithm is to get the coordinates of each pixel of the given calcium wave and save them in the temporary matrix-like object. The resulting file is “waves_inds.pck” file.
- Generate_metadata – the goal of the task is to create a tabular dataframe object to store a coordinate of each pixel of the given calcium wave, together with its intensity. Additionally, for each calcium wave, extreme points, together with Euclidean center and center of mass points are calculated for further analysis.
- Cleanup – the goal of the task is to delete any temporary files created in the analysis

1. 3_find_neighbours:

- Find_neighbours – the task searches for the neighbours of each calcium wave by creating a bounding box, defined by the tolerance_xy and tolerance_t values specified by the user. The center of the bounding box is located at the Euclidean center of the calcium wave. For the extreme points of the wave, a bounding box extends in each direction by tolerance_xy and tolerance_t values, accordingly. Any calcium wave located within the range of the bounding box is treated as a neighbour of that calcium wave. The task produces a neighbours.csv file, which lists all the neighbours for the given calcium wave, together with basic information, such as distance between centers of the calcium waves, in xy and t axes respectively. An additional file, named “neighbour_statistics.csv” is also generated, with a summary for each calcium wave.
- Find_repeats – The task is an extension of the previous step, which works sequentially. For each group of neighbours, it checks the intersection between a shape and its neighbours. The calcium waves are projected into an xy plane beforehand. If the Intersection Over Union for the two shapes is greater than Intersect_threshold parameter specified by the user, the calcium waves are considered repeats of each other. As a result, the task assigns a “single” category for single calcium waves, and “repeat” category for repeated calcium waves. Temporary “repeats.pck” and “singles.pck” files are generated.
- Generate_csvs – The task produces final two csv files for repeated calcium waves and single calcium waves. Some morphological features are calculated for each object (for repeats, those features are averaged across all repeated instances).

### Usage


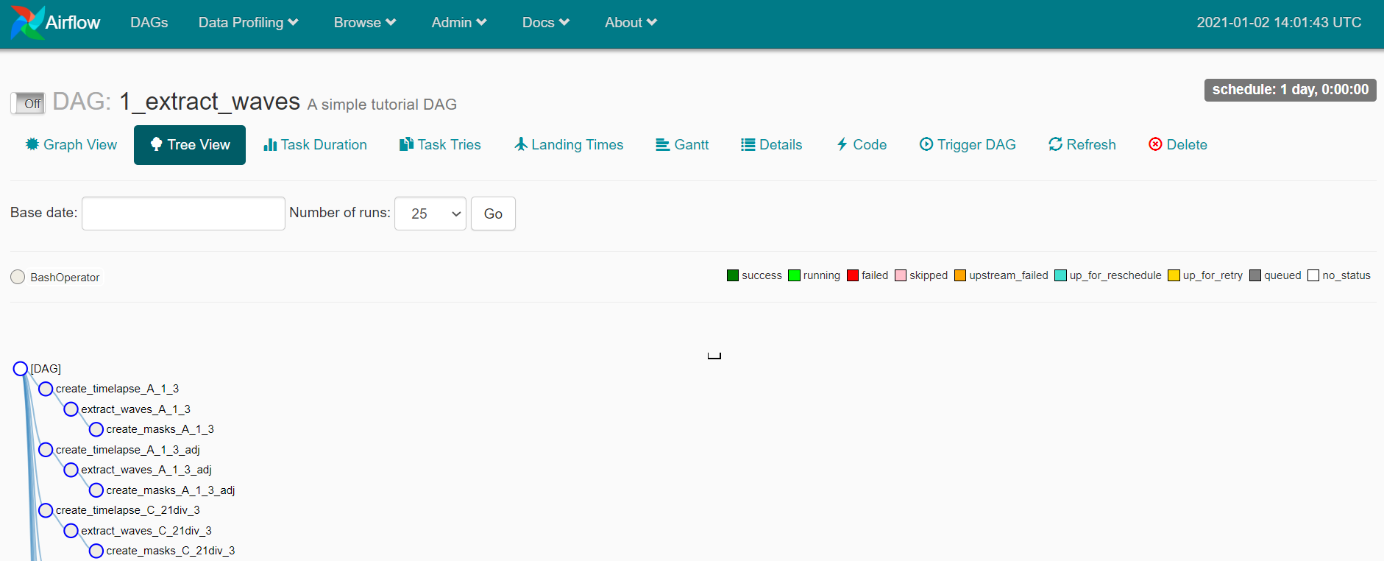


For each file, a set of tasks described in [Pipeline](#_Pipeline) is created. The tasks are executed sequentially. It is also possible to visualize task in a Graph-like manner by clicking “Graph View” tab.

To run a DAG, it is necessary to first launch it by changing its state using a ON/OFF switch in the top left position in the image:


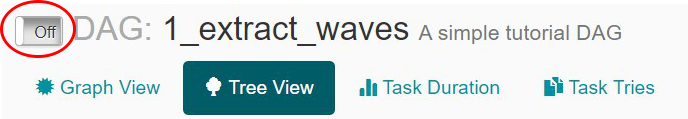


Once clicked, the switch changes its state to On, and the DAG will start executing the tasks.


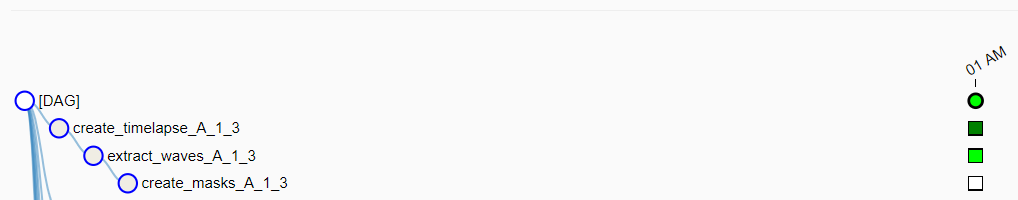


the green circle at the top-right corner indicates that DAG is running and there are remaining tasks to be executed. The dark green box next to the “create_timelapse_A_1_3” task indicates that the task of creating timelapse for the file “A_1_3” has ended with success. The light green box next to the “extract_waves_A_1_3” indicates that the task is currently running. Blank boxes indicate that the task has not started yet.

The full legend of colors is listed on the top of the DAG scheme:


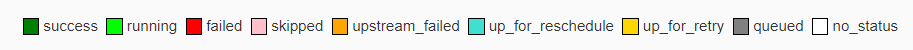


It is possible to stop a running DAG by clicking into the instance circle at the top of the task’s status list:


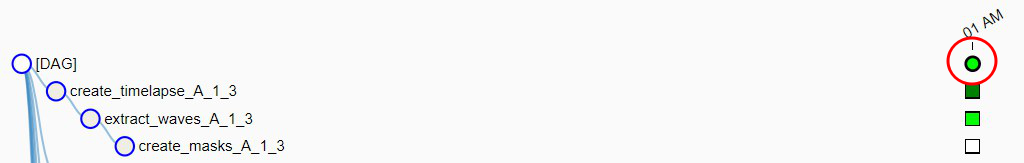


Once clicked, a menu will show with an option of marking a DAG as failed or completed successfully:


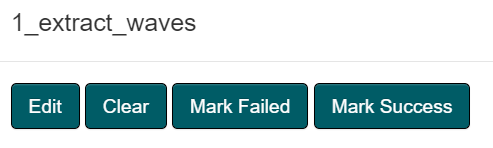


Both options will result in terminating a DAG.

Important

In case a DAG needs to be executed again, a Trigger DAG button should be clicked. Switching off and on will not invoke the DAG again. However, to trigger a DAG, it is necessary to leave a DAG in the ON mode.


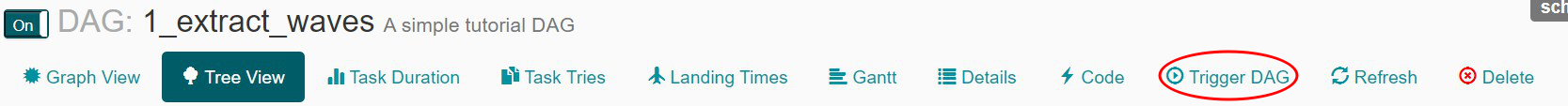


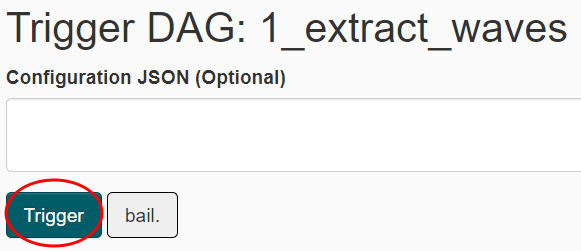


If during execution new files have been added and are not visible in the DAG’s task tree, it is possible to refresh the DAG by using the Refresh button:


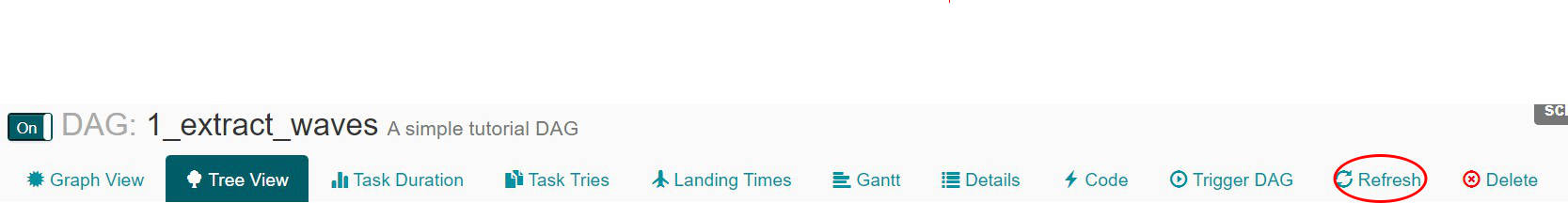


After refreshing, all new files should be visible in the DAG’s tree.

For each file processed in the Airflow application, a directory in a data folder specified in docker-compose.yml is created automatically:


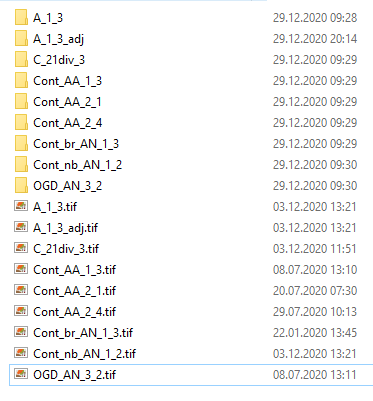


All files generated in the processing pipeline will be saved in these folders.


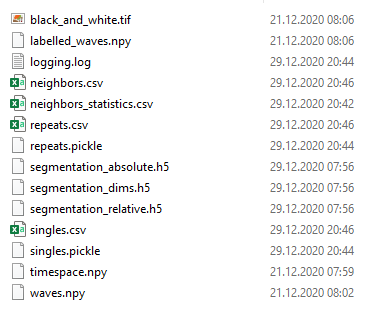


These files are required for the processing pipeline as well as for Streamlit application to run properly.

### Airflow configuration

Airflow provides a built-in functionality of configuring the pipeline for a specific user. A table with short description of each customizable parameter is provided below. A full description of the parameters is listed later in the manual.

| Parameter Name | Description | Default Value |
| --- | --- | --- |
| [Filename](#_Filename) | A filename of a timelapse sequence to be processed from the data directory. Leave ‘all’ if all the files should be processed | All |
| Intersecion_threshold | How much should the calcium waves overlap in their z-projection to be treated as repeats. | 0.8 |
| [Grain_size](#_Standard_deviation_height_range) | How many adjacent pixels in height dimension to take into account during std calculation | 1 |
| [SD_theshold](#_Standard_deviation_threshold) | Standard deviation threshold for detecting calcium events | 5 |
| [Tolerance_t](#_Tolerance_t) | A maximum distance between subsequent calcium waves to treat them as neighbours | 4 |
| [Tolerance_xy](#_Tolerance_xy) | A maximum distance in xy plane to treat two different calcium waves as neighbours | 20 |
| [Use_watershed](#_Use_watershed) | Whether during mask generation, a Watershed algorithm for splitting adjacent calcium waves should be used | 0 |
| [Volume_threshold](#_Volume_threshold) | Volume threshold in total number of pixels to treat a shape as a calcium wave. Calcium waves below volume_threshold will be discarded from the analysis. | 45 |

Those parameters can be set in the Variables section in the Admin tab:


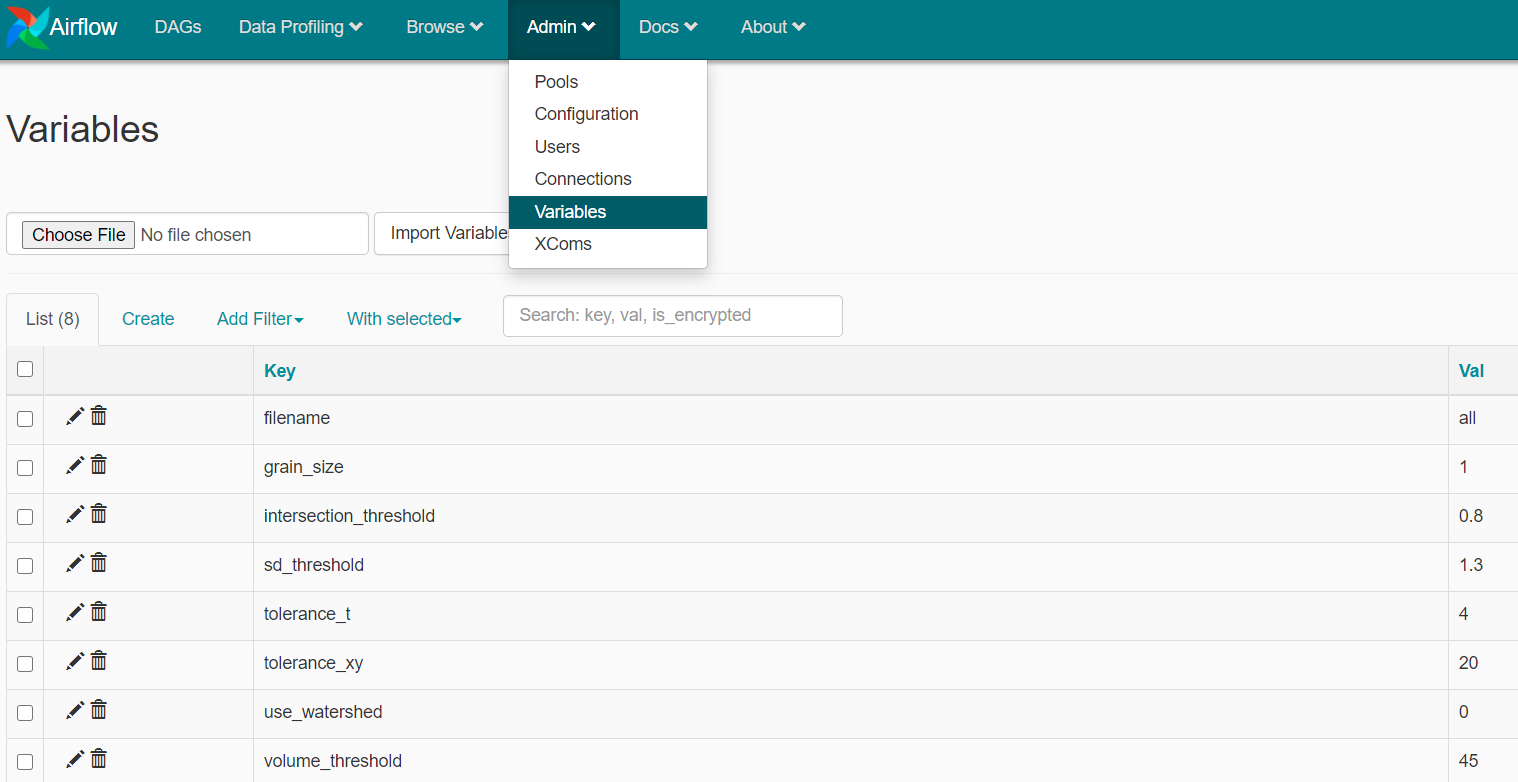


To change the parameter value, you may use a Pen icon next to the parameter name.

Important

It is possible to override the default parameter values. These values are loaded from the **variables.json** file, located in the parent directory of the application. Modifying the values inside the **variables.json** file will change the default values of the application from the next startup.

#### Filename

This parameter specifies the filename of the image series to be processed. If all the images inside the data directory should be analyzed, a special value for the parameter has been defined. To process the files, the “all” value should be entered.

#### Intersection_threshold

Defines, what is the threshold for the Intersection Over Union between projections of the two calcium waves to consider those waves as repeats.


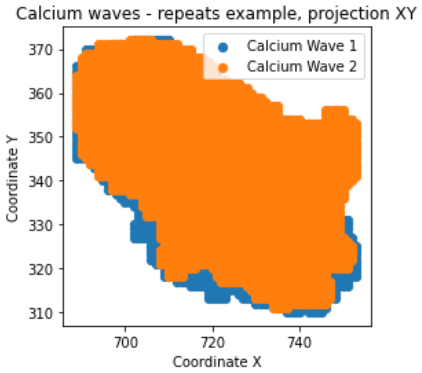


In the above example, the projections of the two calcium waves heavily overlap with each other. The value of Intersection Over Union is high in this case, and if is greater than Intersect_threshold, they will be treated as repeats.


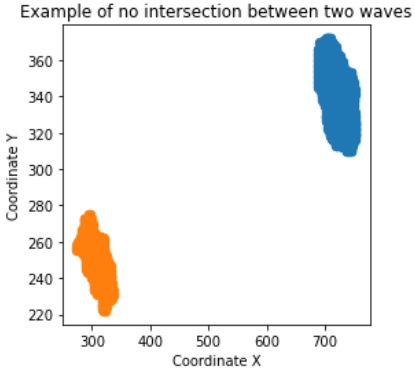


In the above case, the Intersection Over Union is equal to 0, as there is no overlap between the waves. Unless the Intersect_threshold is set to 0, these waves will not be treated as repeats.

#### Grain_size

This parameter specifies, how many consecutive pixels in the image should be taken into account during thresholding. The thresholding is the process of extracting signalling events from the image series. The parameter is used in ‘extract_waves” task in “1_extract_waves” DAG.

#### Standard_deviation_threshold

The threshold value for the standard deviation, above which a pixel is treated as calcium event. Pixels with values below the standard deviation threshold will be treated as background.

#### Tolerance_t

This parameter is used in the “find_neighbours” task in the “3_find_neighbours” DAG. This parameters specifies, how far calcium waves can be separated in terms of the coordinate to be considered neighbours. For example:


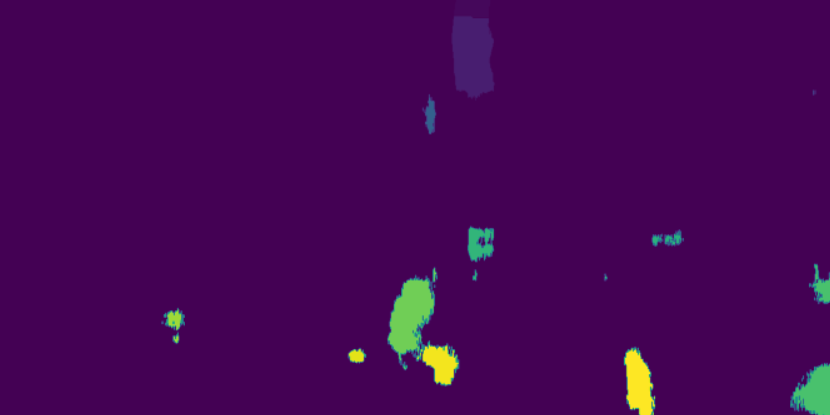


For a green shape, a bounding box with respect to the t coordinate is drawn:


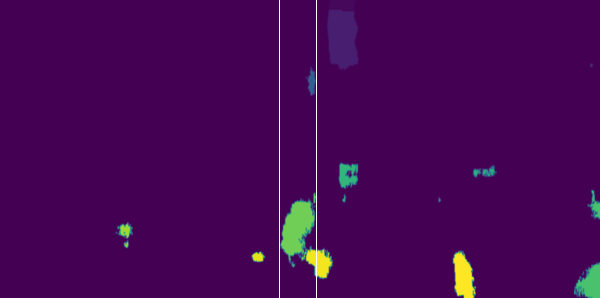


A bounding box is enlarged by tolerance_z parameters from both sides:


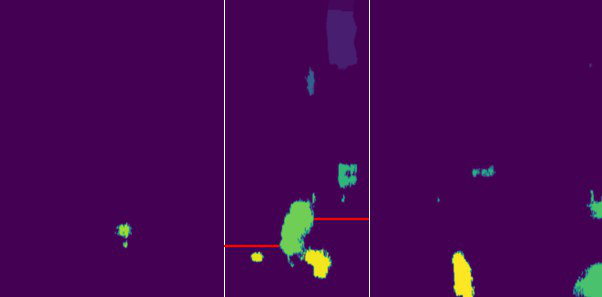


The red lines’ length is specified by the tolerance_z parameter. Any calcium wave that is contained inside the defined region, is considered a neighbour.

#### Tolerance_xy

This parameter is used in the “find_neighbours” task in the “3_find_neighbours” DAG. This parameter specifies, how far calcium waves can be separated in terms of x and y coordinates to be considered as neighbours. For example:


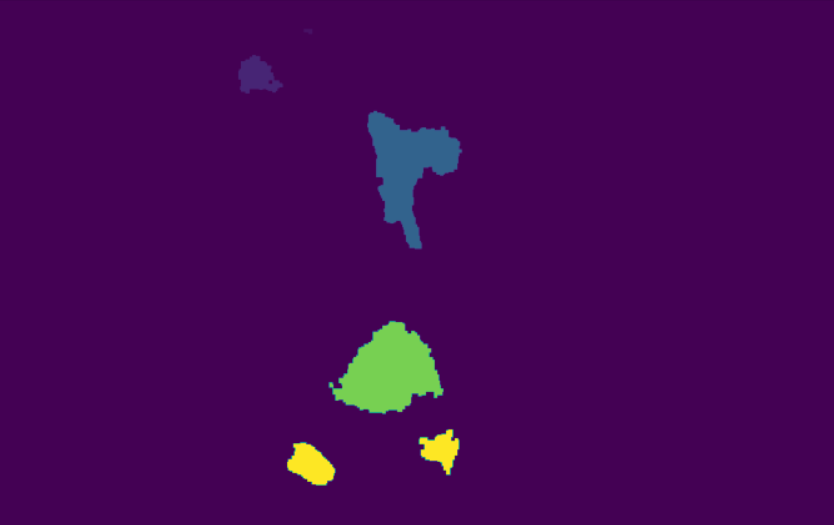


In the image, Astral has detected multiple calcium waves, marked with distinguishing colors. For a given calcium wave, a bounding box is drawn:


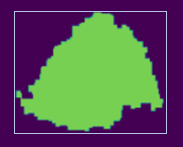


This bounding box is expanded in both x and y directions – the tolerance_xy determines how far should the box be expanded:


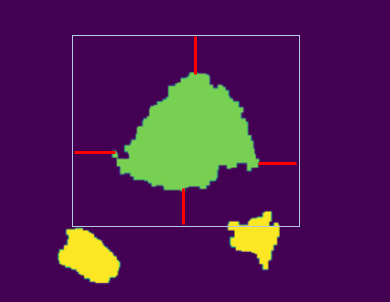


The red lines indicate the size of the enlargement of the bounding box – each of those lines expanded the bounding box by tolerance_xy parameter. In other words, the tolerance_paramer has enlarged the bounding box by twice the tolerance_xy value in both x and y coordinates. Any shape that either crosses the region defined by the bounding box is considered a neighbour. In the case above, one of the waves is a neighbour, as it crosses the boundaries, whereas the other one is not considered a neighbour.

#### Use_watershed

This parameter is used in the “Create_masks” task in the “1_extract_waves” DAG. If the parameter is set to 1, the task additionally uses a Watershed algorithm for splitting adjacent waves apart. If the parameter is set to 0, the task does not perform this additional step. Below you may find an example of a 2D scenario for a Watershed algorithm:


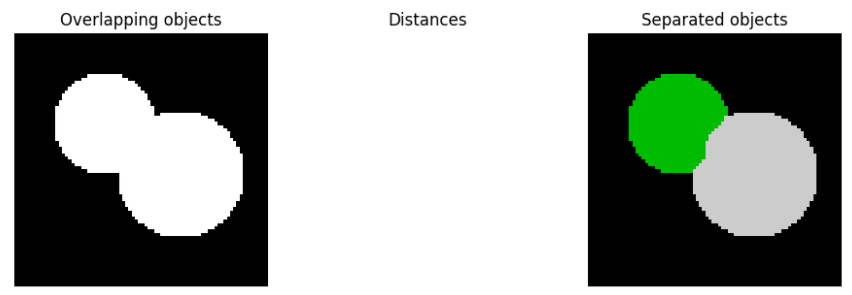


Figure 1. Splitting of overlapping objects. Source: <https://scikit-image.org/docs/dev/auto_examples/segmentation/plot_watershed.html>

The algorithm works in 3D scenario. Basing on intensity of the pixels forming a calcium wave, it determines whether a case contains multiple waves joined together.

#### Volume_threshold

The Volume threshold is used in the “label_waves” task in the “2_segment_waves” DAG. It drops calcium waves, which consist of less pixels than volume_threshold. If the sum of all wave pixels (across all timesteps) is less than the threshold, it is disregarded from further analysis.

## Streamlit application

### Overview

The purpose of Streamlit is to deliver a simple, but powerful solution for visualization and insight into the processed data. Streamlit provides a useful navigation through the astrocytic timelapse, simplifies the process of localization of particular astrocytic events by tabular visualizations, local filtering and markdown. Furthermore, Streamlit provides a 3D visualization of astrocytic events – a particularly useful feature for dynamics study and configuration troubleshooting.

This simple, yet powerful tool fills a gap in the Airflow processing pipeline, which is a lack of immediate visual navigation through the image series. Using Airflow-generated data, Streamlit provides a link between tabular data and imagination. As tabular data is important for problem statement and experiment interpretation, it does not provide a visual representation for the generated data. Therefore, the intention of Streamlit was to compensate the shortcomings of the processing pipelines, such as Airflow.

### Usage


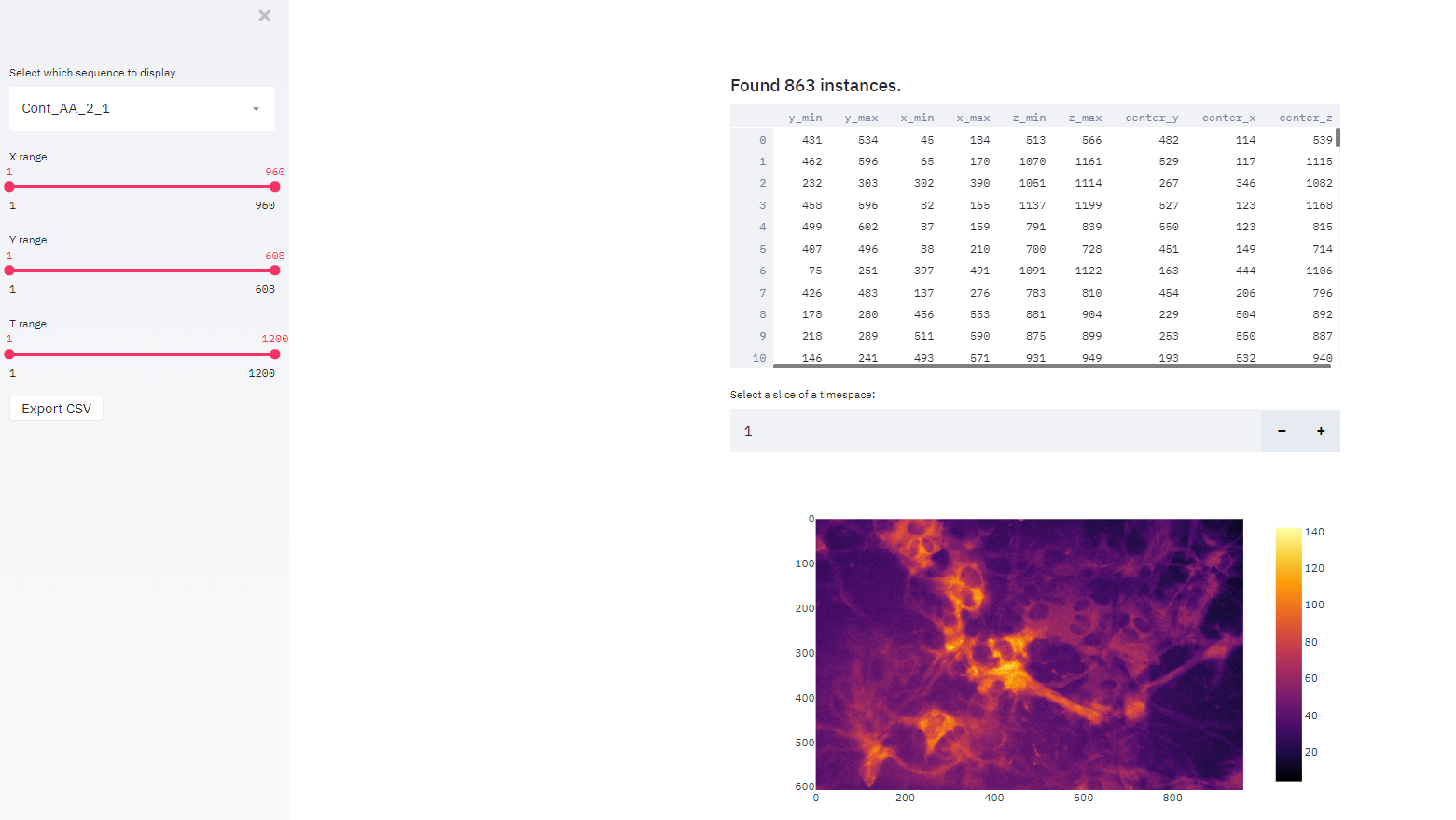


The Streamlit application enables the user to inspect the timelapse in terms of detected calcium waves. First, a list of all calcium waves with their ids and coordinates is shown. To check the location of a given calcium wave in the timelapse, it is possible to navigate through the timelapse as shown here:


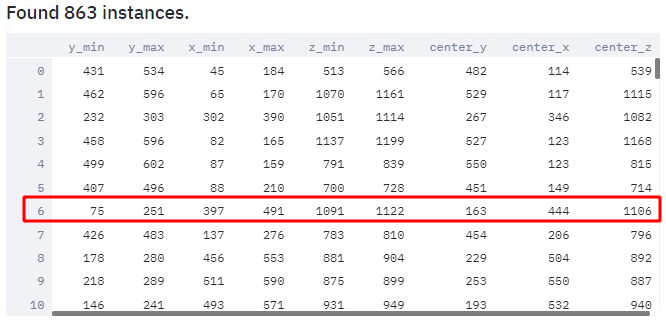


We would like to find the location in the series of the calcium wave with the id of 6:


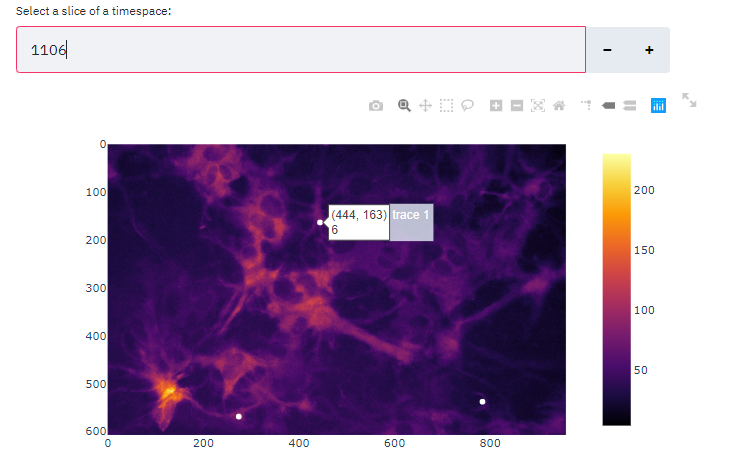


To do that it is necessary to select a slice of a timespace of the center t coordinate of that calcium wave. Then, the calcium wave is marked with a white dot. In this case, two more calcium waves are also present on the slice.

Three sliders on the left can be used to filter the calcium wave list.


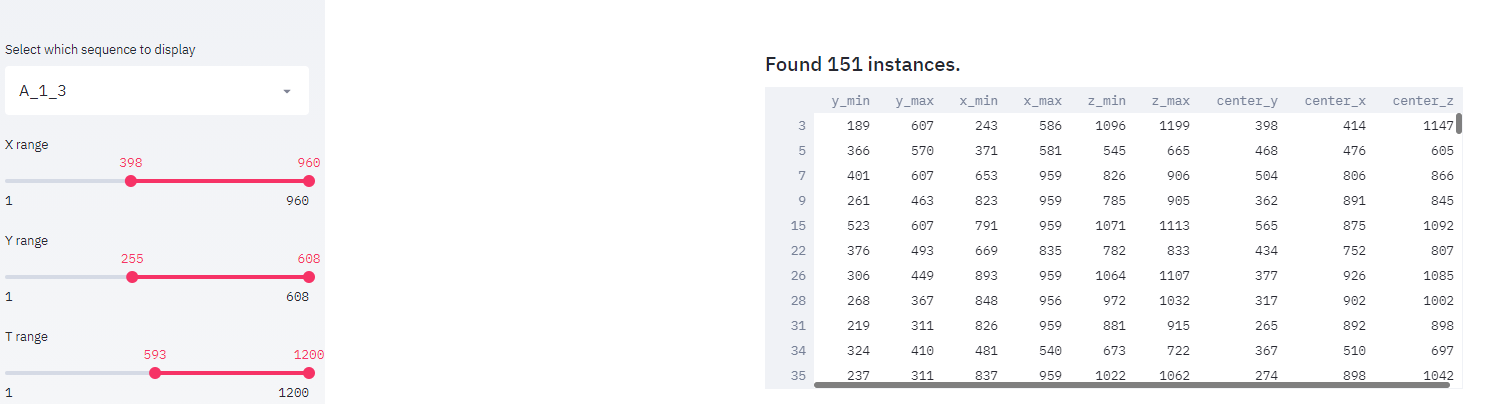


In this case, ranges in all dimensions have been reduced. Only those calcium waves that are contained in this constrained subspace are listed.

It is possible to view any calcium wave in 3D by selecting it by its id:


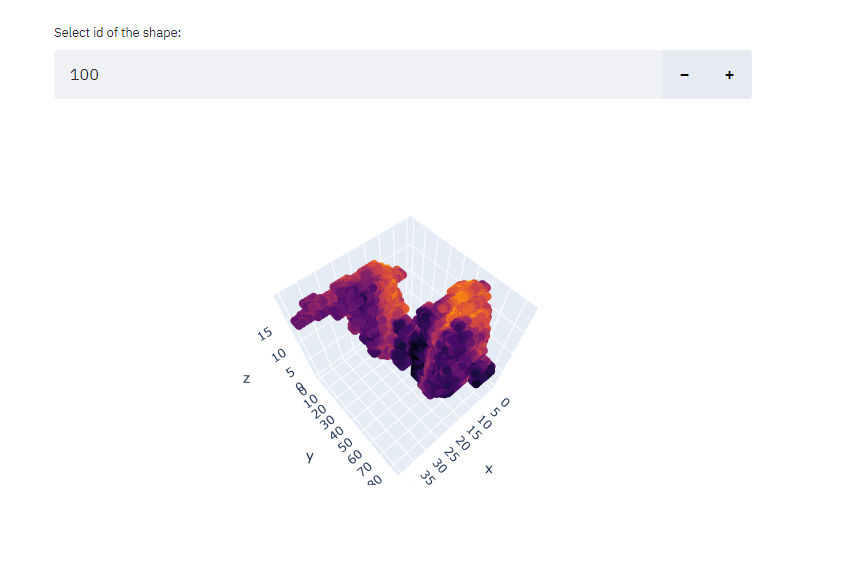


In this case, a calcium wave of id 100 is shown.

Important

The Streamlit application uses files generated in the “1_extract_waves” DAG and the “2_segment_waves” DAG. It is not required to run the “3_find_neghbours” DAG for visualizing the Streamlit application.
