# Supplementary material for: Analysing Intercellular Communication in Astrocytic Networks Using “Astral”
Source: Front Cell Neurosci. 2021 Jun 15;15:689268. doi: 10.3389/fncel.2021.689268 (PMC8239356; doi:10.3389/fncel.2021.689268)
Supplement: Supplementary file 2 [file Data_Sheet_2.DOCX]

**Developer’s guide**

Astral was designed with the intention to easily expand its functionality. The application uses Apache Airflow platform to define jobs, which accomplish a particular task. Currently, in Airflow there are four jobs defined:

- Preprocessing of Carl Zeiss timelapses for intensity and drift correction and converting them to a tiff file format
- Extraction of the calcium events from the timelapses
- Segmentation of calcium events and calculation of the morphological statistics for each event
- Neighbourhood and repeat search for each of the events

Those jobs form a baseline pipeline for the frequential analysis of calcium events. However, the user can define new jobs very easily.

The whole solution follows the standard convention for creating Python PyPI packages. The repository contains the package for defining Directed Acyclic Graphs (DAGs) inside Airflow. DAGs are Python scripts, which define the order and execution of other Python scripts. For reference, see the **astrowaves.airflow.dags** directory to discover the approach to define DAGs. For more detailed description, see the official page https://airflow.apache.org/docs/apache-airflow/stable/tutorial.html

DAGs are composed of tasks, which are Python objects that execute Python scripts. Each task should execute one script defined in the **astrowaves.tasks** package. The convention is to create a separate module in that directory for each task. The whole task should be defined in one, separate module inside the **astrowaves.tasks** package. The task should not be coupled with other task modules. In case the universal functionality could be introduced during the development of the new task, all the functions should be provided in the utility script, which provides common protocols for all the tasks.

To sum up, in case a new DAG should be defined, the module inside the **astrowaves/airflow/dags** folder should be added, which follows the DAG definition for Airflow platform. Each task used inside the DAG should be defined in its own separate module inside the **astrowaves/tasks** folder.

The full specification for installed packages used inside Astral is provided in the **requirements.txt** folder. In case a new package should be installed inside Astral’s environment, it should be added with correct version to that file. The development environment for Astral has been added in terms of Poetry dependency manager. To set up development environment, a virtual environment should be set up with a Python version 3.7. Once the environment has been set up, all the packages should be installed via Poetry.

Astral runs inside Docker containers. In case a custom configuration for the Docker containers should be introduced, **astro-Dockerfile** and **streamlit-Dockerfile** files should be modified for Airflow or streamlit, accordingly.In case a new major functionality should be introduced to Astral, feel free to submit a Change Request in the repository https://github.com/wprazuch/Astral
